# Lipid nanoparticles: a promising tool for nucleic acid delivery in cancer immunotherapy

**DOI:** 10.1007/s12032-025-02939-3

**Published:** 2025-08-06

**Authors:** Tasneem Abaza, Eman E. Mohamed, Mohamed Y. Zaky

**Affiliations:** 1 Biotechnology Program, Institute of Basic and Applied Sciences (BAS), Egypt-Japan University of Science and Technology (E-JUST), New Borg El-Arab City, 21934 Alexandria Egypt; 2 Molecular Physiology Division, Department of Zoology, Faculty of Science, Beni-Suef University, P.O. Box 62511, Beni-Suef, Egypt

**Keywords:** Immunostimulatory nucleic acids, Lipid nanoparticles, Drug delivery, Cancer immunotherapy, Gene therapy

## Abstract

Cancer immunotherapy and nucleic acid therapy have demonstrated significant potential in reshaping cancer treatment paradigms. The combination of these therapeutic approaches offers promising prospects for enhancing systemic anti-tumor effects while minimizing adverse reactions commonly associated with traditional treatments. However, the clinical efficacy of these innovative therapies is often hindered by several delivery-related challenges. These include issues like degradation of therapeutic molecules, limited cellular uptake, the essential requirement for nuclear entry, and risks of off-target toxicity that can negatively impact patient safety. In this challenging landscape, nanoparticle delivery systems, particularly lipid nanoparticles (LNPs), have emerged as a groundbreaking solution to overcome these obstacles. LNPs facilitate the safe and efficient delivery of nucleic acid therapy directly to immune cells, enhancing their bioavailability and therapeutic impact. This review article examines the evolving role of LNPs in the landscape of cancer immunotherapy. Recent advancements in LNP-based nucleic acid delivery illustrate their potential to revolutionize the field by enabling precise modulation of gene expression and immune responses, paving the way for improved cancer treatment outcomes and providing a more effective arsenal against this complex disease.

## Introduction

Cancer immunotherapy has demonstrated tremendous promise, evident in the growing number of immunooncology drug approvals in recent years [1]. Cancer immunotherapy offers a diverse array of strategies including immune checkpoint inhibitors (ICIs), cancer vaccines, CAR T cell therapy, and oncolytic viruses [2].

Nucleic acid therapies including antisense oligonucleotides (ASO), plasmids, immunomodulatory DNA/RNA, small interfering RNA (siRNA), messenger RNA (mRNA), microRNA, and gene-editing guide RNA (gRNA) are appealing because of their versatile capability to influence the target native or synthetic genes expression and regulate immune responses [1]. These versatile attributes are crucial in the development of innovative immunotherapy approaches not only for their potential to modify, either upregulating or downregulating, gene expression [3], but also to regulate immune responses [4, 5].

Several drug delivery approaches were developed to deliver medication to specific areas of the body effectively. These systems not only target the desired sites but also safeguard the active ingredients of the drugs, ultimately improving their efficacy [6]. For many years, nanoparticles (NPs) have been the focus of spacious research studies in drug delivery [6–8]. NPs can enhance drug accumulation in neoplastic tissues by utilizing the increased permeability in tumor arteries. Loading the molecules inside the NP increases the solubility and stability of the drug and inhibits breakdown by serum proteins. Additionally, the release of the drug could be controlled, preventing early leakage [9]. Furthermore, various NP formulations with varying encapsulation efficiencies and zeta potentials are shown, all while keeping a consistently very small size, demonstrating the adaptability of NP employment in this field [Bakhshi et al., 2024] [10].

Specifically, lipid nanoparticles (LNPs) such as nanostructured lipid carriers (NLCs), solid lipid nanoparticles (SLNs), and liposomes have been thoroughly investigated for their effectiveness in delivering hydrophilic and hydrophobic drugs, showcasing promising results for clinical applications [11].

Doxil, the initial FDA-approved nano-drug, consists of doxorubicin (DOX) enclosed within a PEGylated liposome tailored for the treatment of ovarian cancer, breast cancer, and diverse solid tumors [12, 13]. The PEGylated liposomal doxorubicin Doxil presents numerous advantages over free DOX, such as a significant decrease in cardiotoxicity, prolonged presence in the bloodstream, and passive targeting of tumors through utilization of the improved permeability and retention (EPR) effect [12]. Furthermore, the Onpattro approval for treating Amyloidosis in the United States and European Union in 2018 served as robust validation for employing LNPs as a delivery system for nucleic acid drugs. LNPs are increasingly playing a vital role in the expanding domains of mRNA and replicon-based therapies, protein replacement strategies, and facilitating gene-editing approaches [14].

A research study investigated the potential of LNPs encapsulating siRNA to target PCTAIRE1 in tumor cells in vivo. The study showed that treatment with PCTAIRE1 siRNA-lipid nanoparticles effectively reduced the expression of PCTAIRE1 for up to four days, leading to reduced tumor volume and weight in colorectal cancer and melanoma cells compared to the control group. The therapy increased apoptosis rate in the tumor cells, demonstrating the potential of siRNA-lipid nanoparticles as an innovative therapeutic strategy for targeting cancer cells [15].

In another study, a new method was devised using LNPs to transport nucleoside-modified mRNA and the adjuvant monophosphoryl lipid A (MPLA) for therapeutic cancer vaccination. The nucleoside-modified mRNA improves translation efficiency and safety by preventing the activation of type I interferons (IFNs), that are accountable for stimulating and T-cells and antigen-presenting cells immunity. Through the simultaneous delivery of the modified mRNA with MPLA, the research team achieved heightened expression of antigen in living organisms while replacing the type I IFN reaction with a more manageable adjuvant. This approach is designed to bolster efficient antigen-specific T-cell immunity and improve the safety standards of mRNA vaccines targeted for cancer treatment [16]. To address these advancements, the following sections of this review will focus on key topics that highlight the evolving role of LNPs in nucleic acid delivery for cancer immunotherapy. We begin by exploring the structural and functional features of LNPs that make them suitable for delivering various nucleic acid-based therapeutics. Next, we discuss specific examples of LNP formulations that have demonstrated success in preclinical and clinical settings, including siRNA, mRNA, and gene-editing approaches. Finally, we examine the underlying mechanisms by which these systems enhance immune activation and anti-tumor responses. By emphasizing both the technological innovations and therapeutic impact, this review aims to provide a comprehensive understanding of the significance and potential of LNPs in advancing cancer immunotherapy.

## Lipid nanoparticles: an overview

In the late 1970s, early efforts to encapsulate nucleic acid in liposomes encountered challenges with poor encapsulation and passive encapsulation procedures, impacting their effectiveness [17–19]. Also, the utilization of constitutively charged cationic lipids in lipoplexes led to issues with potency and tolerability, limiting their clinical utility. The emergence of ionizable cationic lipids represented a significant advancement in lipid formulations, offering pH-titratable properties that enable favorable interaction with the endosomal membrane while addressing issues of quick elimination and low tolerability. These ionizable lipids are frequently paired with PEGylated lipids to aid in the process of formulation, enhance vial stability, and optimize pharmacokinetics and biodistribution. Although PEGylation disrupts cellular uptake, a critical design improvement involving the utilization of PEGylated lipids that enhance cellular uptake has played a key role in the success of the present generation of LNPs [14, 20].

## Ionizable cationic lipids

The use of ionizable lipids in LNPs marks a pivotal departure from permanently charged cationic lipids, exerting a substantial influence on LNPs potency and performance. Their ionizable nature facilitates the creation of particles encapsulating payloads, resulting in enhanced pharmacokinetics, prolonged bloodstream half-life, and improved accumulation in specific tissues like solid tumors. Moreover, in contrast to stable positively charged lipids, the ionizable nature of these lipids improves the overall tolerability profile, mitigating the risk of cellular toxicity, immune system activation, and blood aggregation [20].

The design of ionizable lipids is crucial for creating effective LNPs for drug delivery. After the disclosure of DODAP by Semple et al*.* [20], researchers embarked to investigate this area. An axial advancement was the finding that rising the unsaturation level in the ionizable lipid hydrophobic part significantly boosts its effectiveness [21]. Prior to this, DODAP, like many cationic lipids, utilizes oleyl groups, which is a C18 carbon chain with a single cis-double bond. Research by Heyes et al*.* [21] demonstrated that having more double bonds in close proximity, such as the linoleyl group, enhanced endosomal fusion and delivery by promoting the formation of the fusogenic hexagonal II (HII) phase within the lipid particle. Subsequently, the development of the DLinDMA lipid enabled the initial successful demonstration of RNA interference in primates through a systemically administered LNPs [22].

*Semple *et al*.* initiated a novel study to further explore the design of ionizable lipids and LNPs platforms, revealing additional crucial findings and show the significance of LNPs composition adjustments, such as reducing PEG content and increasing ionizable lipid content, which resulted in a remarkable five times increase in potency without altering the components of lipid [23].

These findings have influenced the compositions of current products like OnpattroTM and various mRNA-based formulations undergoing clinical evaluation, characterized by similar molar ratios of PEG-lipi at 1.5% [14]. The refinement of structural features led to the development of DLin-MC3-DMA. In a comprehensive SAR screening, Jayaraman and colleagues clearly highlighted the significance of maintaining a specific pKa range between 6.2 and 6.5 for optimal activity in hepatocytes [24]. Furthermore, they illustrated that by substituting the ketal linker with a carboxylic ester while preserving the single carbon attachment point for linoleyl chains from KC2, additional improvements in potency could be achieved. As a result, the MC3 lipid, which stemmed from these findings, is the ionizable lipid featured in the Onpattro drug [24, 25].

Biodegradable LNPs containing ionizable lipids can be broken down by enzymes [26, 27]. In contrast, MC3, a key component of LNPs, is non-biodegradable and displays a half-life of around 70 h in serum. While MC3 has demonstrated safety and tolerance at specific doses in Onpattro, the biodegradability of LNPs becomes particularly important for applications requiring more frequent administration, such as mRNA delivery for therapeutic protein production [14]. In such cases, dosing frequency will depend on transcript turnover and protein half-life, potentially requiring more frequent dosing compared to siRNA [27, 28]. Given the widespread use of ionizable lipids in LNPs products, it is essential to prioritize the redesign of these lipids to exhibit biodegradable properties. This can be achieved by introducing carboxylic ester groups that can be split by esterases. Some newly developed biodegradable lipids have shown greater potency than MC3, with certain compounds yielding up to fivefold higher protein expression, as reported by *Sabnis *et al*.* [27].

## PEGylated lipid

PEG-lipids play various important roles in LNPs. Their structure comprises a hydrophilic PEG-polymer attached to a hydrophobic lipid anchor. Positioned on the surface of lipid particles, they historically extended circulation time by acting as a steric barrier against plasma proteins, enhancing particle accumulation in disease sites like solid tumors. These functions were analogous in earlier stabilized plasmid-lipid particles (SPLP) utilized for gene therapy involving plasmid DNA delivery [29–31].

In contemporary LNPs formulations, PEG-lipids have been modified to achieve a balance between circulation time and cellular uptake, and to influence particle size during manufacturing. The absence of PEG-lipids during the LNPs manufacturing process could lead to particle fusion due to the ethanolic environment low pH. However, the steric barrier provided by PEG-lipids prevents this, ensuring the production of homogeneous particles with narrow size distribution, typically ranging from 50 to 100 nm, based on the specific quantity of PEG-lipid utilized [14].

Although PEG-lipids are beneficial in inhibiting undesired opsonization in the blood circulation, they can impede critical processes involved in successful payload delivery and activity in older lipid particle applications. They hinder the binding of ApoE to the LNPs surface required for hepatocyte delivery and impede endosomal escape crucial for intracellular payload delivery [32, 33]. Therefore, to address these issues, the amount of PEG is kept as low as possible, which, along with elevated ionizable content, has been associated with a reported fivefold improvement in potency [23].

PEG-lipids could be designed to disengage from the nanoparticle, and the rate at which they do so is a critical factor. This rate could be altered by modifying the PEG-lipid anchor size. Originally used in oncology liposomal systems, adjusting the anchor size was found to create particles that circulated in the blood for extended durations [29, 30, 34]. A detailed characterization of PEG-lipid detachment rates using dual labeled LNPs has been conducted to measure and correlate them with pharmacokinetics and biodistribution [14].

The rate of PEG diffusion is critical for safety. Permanently attached PEG-lipids, leading to longer particle circulation, have been linked to inducing an immune response when combined with nucleic acid payloads. This prompts a robust, enduring antibody reaction against the particle’s surface chemistry, particularly the PEG component, leading to expedited removal from the bloodstream and acute hypersensitivity upon subsequent administrations [35, 36].

In addition to PEGylated and ionizable lipids, several other classes of lipids play pivotal roles in the structure and function of LNPs for nucleic acid delivery, including anionic, cationic, amphiphilic, hydrophilic, and hydrophobic lipids. Each type contributes uniquely to the encapsulation, transport, and release of nucleic acids. Cationic lipids (e.g. DOTAP, DDA) possess positively charged head groups that interact electrostatically with the negatively charged phosphate backbone of nucleic acids, promoting strong encapsulation and complex formation. However, their permanent charge can lead to cytotoxicity and limited biocompatibility. Ionizable lipids, by contrast, remain neutral at physiological pH, reducing toxicity, and become protonated in acidic endosomal environments to facilitate endosomal escape. Anionic lipids, though less commonly used alone due to electrostatic repulsion with nucleic acids, can be incorporated to modulate membrane fluidity and fusion properties. Amphiphilic lipids, possessing both hydrophilic and hydrophobic domains, support the bilayer formation and stability of LNPs, while assisting in the self-assembly process. Hydrophobic lipids, often forming the core or internal domain, contribute to structural integrity and can aid in sustained release. Hydrophilic lipids, such as PEGylated lipids, provide a stealth layer that enhances systemic circulation by minimizing opsonization and clearance by the reticuloendothelial system (RES).

The encapsulation mechanism typically involves the rapid mixing of lipids dissolved in ethanol with nucleic acids in an aqueous buffer, leading to spontaneous nanoparticle self-assembly through electrostatic and hydrophobic interactions. During systemic circulation, LNPs protect the nucleic acids from enzymatic degradation and promote cellular uptake, often via endocytosis. Upon internalization, the acidic environment within endosomes protonates ionizable lipids, destabilizing the endosomal membrane and facilitating cytoplasmic release of the payload. Each lipid component thus plays a strategic role in optimizing encapsulation efficiency, biodistribution, and therapeutic efficacy of nucleic acid-based therapies.

Despite their promising applications, LNPs face several limitations that must be addressed for broader clinical translation. One notable challenge is the immunogenicity associated with PEG, commonly used to enhance circulation time and stability of LNPs. Repeated administration of PEGylated LNPs can lead to the development of anti-PEG antibodies, potentially reducing efficacy and causing adverse immune reactions. Additionally, scalability and reproducibility of LNP production remain significant hurdles for industrial manufacturing, impacting batch-to-batch consistency and cost-effectiveness. To mitigate these issues, alternative polymers such as polysarcosine and zwitterionic lipids are being explored as PEG substitutes to reduce immunogenicity. Moreover, biodegradable lipids and ionizable lipids are being developed to enhance safety and facilitate efficient clearance from the body. Advances in microfluidic and continuous manufacturing technologies are also improving scalability and quality control, thereby accelerating the translation of LNPs from bench to bedside. As summarized in Table 1, various types of LNPs exhibit distinct structural and functional properties that facilitate efficient nucleic acid delivery. Each system offers unique advantages in terms of stability, encapsulation efficiency, and intracellular trafficking.

**Table 1:** Overview of lipid-based nanoparticles for nucleic acid delivery: roles, advantages, and representative studies

LNPs type	Role in delivery of nucleic acid	Key advantages	Selected References
Cationic Liposomes	Bind negatively charged nucleic acids via electrostatic interaction	High encapsulation efficiency, promotes cellular uptake	Zhang et al. [37]
Ionizable LNPs	pH-responsive release in endosomes; effective cytosolic delivery	Reduced toxicity, efficient endosomal escape	Kulkarni et al. [38]; Hou et al. [39];
SLNs	Physically encapsulate nucleic acids; sustained release	Biocompatibility, structural stability	Mukherjee et al. [40]; Beloqui et al. [41]
NLCs	Load both hydrophilic and lipophilic payloads; improved nucleic acid entrapment	Enhanced stability, loading capacity	Patil-Gadhe et al. [42]; Almeida et al. [43]
PEGylated Liposomes	Extend circulation time; prevent immune clearance	Improved pharmacokinetics, reduced RES uptake	Immordino et al. [44]; Barenholz [45]
Amphiphilic Lipid Systems	Aid self-assembly and membrane fusion with endosomal membranes	Facilitates intracellular delivery	Zununi Vahed et al. [46]
Hybrid Lipid-Polymer NPs	Combine lipid membrane with polymeric core for nucleic acid loading	Controlled release, tunable properties	Liu et al. [47]; Zhang et al. [48]

## Phospholipids and cholesterol

Since the advent of LNPs for siRNA and their recent application for mRNA, phospholipids and cholesterol have been essential elements in LNP formulation. These compounds play a vital role in ensuring the structure integrity, phase transition behavior, and the particles fusogenicity. They are essential for proper encapsulation of the nucleic acid payload and for maintaining long-term stability. Moreover, phospholipids facilitate the formulation process through tangential flow ultrafiltration (TFU). Some research indicates that the specific phospholipid used could significantly impact the effectiveness, although the overall outcome may be influenced by other lipids in the system [14, 49]. A SAR study explored the effects of cholesterol analogs on gene transfection in LNP, revealing that the inclusion of C-24 alkyl phytosterols significantly improved transfection efficiency [50].

## Types of LNPs

**Fig. 1:**
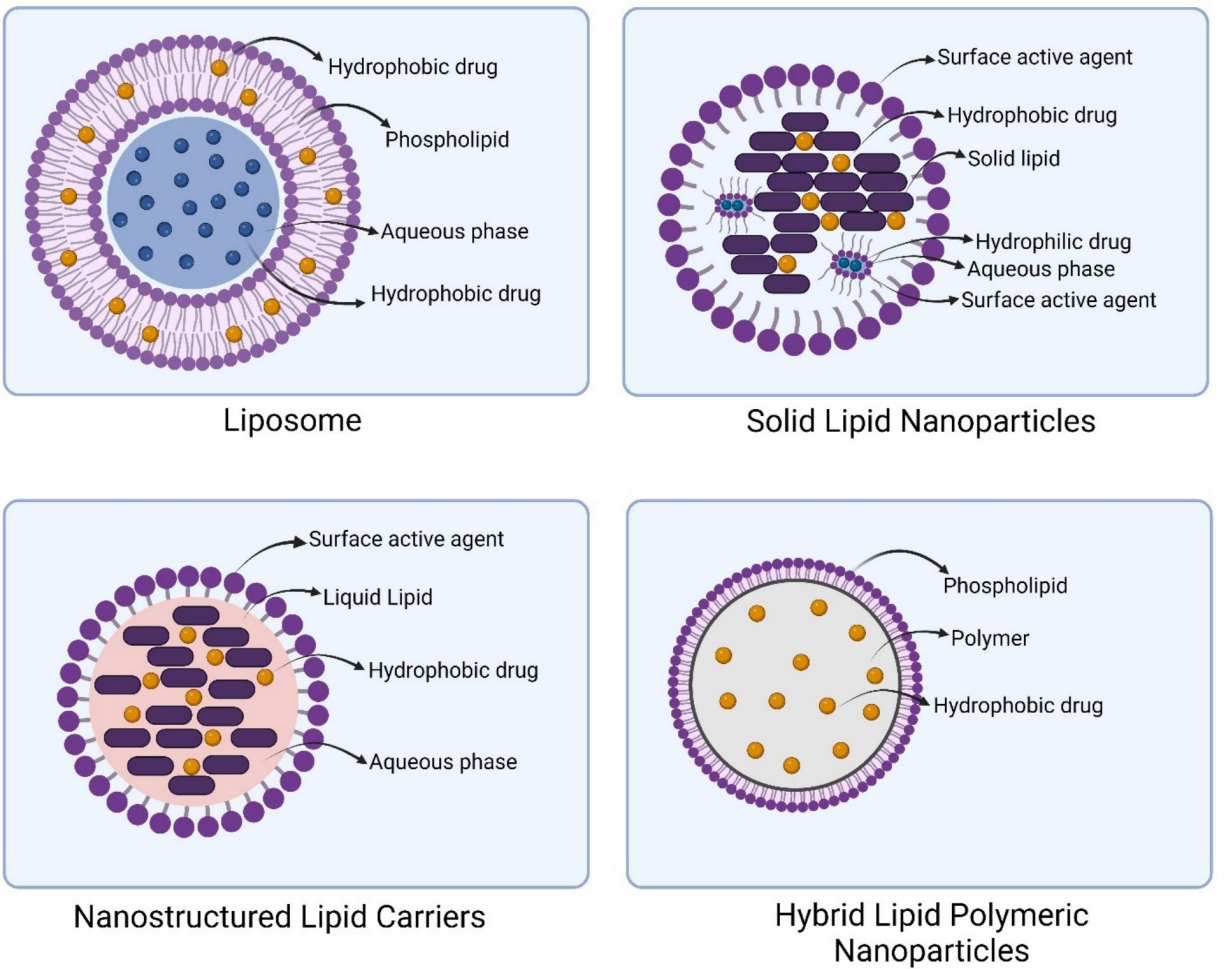
Types of lipid nanoparticles

Lipid-based nanoparticles have arisen as a favorable option for drug delivery due to their low toxicity in vivo, particularly in DNA/RNA and drug delivery applications [51]. They are typically formulated using physiological lipids, known for their biocompatibility and biodegradability [52], and they demonstrate low chronic and acute toxicity [53, 54]. LNPs have emerged as a promising modality in cancer treatment. Comprising biodegradable lipids, LNPs offer a solution to the constraints associated with intravenous delivery, exhibiting enhanced drug bioavailability when administered orally due to their preferential lymphatic system uptake [55]. Based on their nanostructure, LNPs can be categorized into four systems: liposomes, SLNs, NLCs, and hybrid lipid-polymeric nanoparticles (Fig. 1).

### Liposomes

Liposomes are spherical formations made up of an amphipathic phospholipid bilayer surrounding a central aqueous core [56]. The lipid molecules utilized in liposomes are fundamental structures consisting of a head group and hydrophobic hydrocarbon tails linked together by a backbone connector like glycerol [57]. This core–shell nanostructure of liposomes allows them to effectively encapsulate both hydrophilic and hydrophobic molecules. Usually, hydrophobic drugs are encapsulated within the lipophilic layers of the shell [58], while hydrophilic drugs are contained within the aqueous core [59].

Cationic lipids typically acquire a positive charge due to the amines located in the group of polar heads. The existence of positively charged amines enhances the interaction with anions, including those found in DNA. The resulting liposome is formed through a combination of van der Waals forces and electrostatic interactions with the DNA, influencing the shapes of the liposomes [60]. Given the polyanionic property of DNA, cationic (and neutral) lipids are commonly employed for gene delivery, while the utilization of anionic liposomes has been predominantly limited to delivering other therapeutic macromolecules [61].

#### Liposomes: preparation and applications

Liposomes could be prepared through different methodologies, selected based on criteria like the physicochemical properties of liposome components and the loaded drug, the toxicity and concentration of the substance, the medium in which liposomes are dispersed, additional application/delivery processes, desired size and stability, and production costs [62–64]. Common techniques for preparing liposome include thin-film hydration [56, 65], reverse-phase evaporation, solvent-injection [66–68], detergent-depletion [65, 69], and dehydration–rehydration methods [70].

Post-formation processing steps such as sonication [71], extrusion [72–74], and high-pressure homogenization [75] are necessary for achieving desired liposome properties. Furthermore, Industrial-scale production methods such as heating, spray-drying, freeze-drying, and microfluidic-based techniques have broadened the array of preparation options [76].

The microfluidic-based method is an evolving technology enabling precise control over the lipid-hydration process during liposome synthesis [77]. Real-time monitoring technologies such as organic electrochemical transistors (OECTs) hold promise for sensitive monitoring of liposome dynamics [78]. Characterization techniques assessing liposome shape, size, lamellarity, and surface features, including dynamic light scattering, electron microscopy, and size-exclusion chromatography, are crucial for ensuring in vivo and in vitro and performance. These distinct techniques provide ways to estimate liposome properties comprehensively prior to application [79].

## Solid Lipid Nanoparticles (SLNs)

SLNs represent a colloidal drug delivery system that exhibits a spherical morphology and falls within the particle size range of 10–1000 nm. In the mid-1990s, SLNs were first developed by utilizing lipids characterized by melting points exceeding both physiological and environmental temperatures, including fatty acids, triglycerides, and waxes [59, 60].

SLNs are known by their structured incorporation of drugs within a highly organized crystalline framework, alongside emulsifiers. The surfactant is employed to prevent the aggregation of SLNs to maintain stability. Additionally, a co-surfactant is included to enhance the concentration of micelles. Optimizing the surfactant concentration is crucial for stabilizing both the NPs and microparticles, as well as reducing particle size by minimizing aggregation [80, 81].

SLNs provide numerous advantages, including improved nanoparticle stability, efficient drug shielding, controlled release, and the ability to customize properties by altering lipid components [82]. Nevertheless, SLNs encounter some significant challenges: inadequate prolonged drug retention and limited capacity for drug loading. Throughout storage, the lipid matrix undergoes a change in polymorphism from a high-energy state to a low-energy state, resulting in the development of a more structured crystalline lattice and the gradual release of the encapsulated drugs. This polymorphism notably restricts the drug loading capacity, particularly for highly purified lipids [83, 84].

There are three different models that can be employed to classify SLNs depending on the distribution of active ingredients. The methods are drug-enriched core model, drug-enriched shell model, and solid solution model [83, 85]. The formation of SLN could be carried out by four methods: solvent-diffusion evaporation method, membrane contactor method, ultrasonication method, and high-pressure homogenization method, which branches into two subcategories, hot homogenization and cold homogenization. Non-uniform particle sizes resulted from high pressure and ultrasonication methods, whereas the solvent-diffusion evaporation method and membrane contactor method yielded particles of uniform size [80].

The drawbacks of the membrane contactor methods and solvent-diffusion evaporation method include the potential toxicity of organic solvent residue if not fully evaporated from the SLNs, along with the high cost associated with the membrane contactor equipment used in SLN formulation. In contrast, the high-pressure and ultra-turrax homogenization methods are considered safer alternatives relative to the solvent-diffusion evaporation method [80].

## Nanostructured lipid carriers (NLCs)

NLCs represent drug delivery systems that consist of a core matrix comprising both solid and liquid lipids [86]. NLCs, as an advancement in SLN technology, represent the second generation of SLNs. They were refined by replacing some of the solid lipid components in SLNs with liquid lipids, leading to an increased capacity for drug incorporation [87]. Studies have demonstrated that NLCs offer various benefits for drug therapy compared to traditional carriers. These advantages include enhanced solubility, improved storage stability, reduced adverse effects, prolonged half-life, increased permeability and bioavailability, targeted delivery to specific tissues, and reduced adverse effects [86].

The structure of LNPs is characterized by three types of NLCs, namely amorphous type, multiple type, and imperfect crystal type [85]. The imperfect crystal type of NLCs utilizes a highly disordered matrix achieved through blending spatially distinct lipids, resulting in a high drug loading capacity. Nonetheless, this approach tends to have a relatively low encapsulation efficiency (EE) due to the limited drug solubility in solid lipids. In contrast, many NLCs incorporate a higher proportion of oil in conjunction with a solid lipid triggering phase separation. This separation leads to the formation of oily nano-compartments that encapsulate the drug and enhance drug solubility, thereby boosting EE. Amorphous NLCs feature a structureless solid matrix composed of specific lipids like hydroxyl stearate, hydroxyl octacosanol, and isopropyl myristate to prevent crystallization-induced drug leakage [58, 88].

NLCs are prepared using various methods including high-pressure homogenization (HPH), film ultrasonication, emulsification-ultrasonication, solvent diffusion, solvent emulsification evaporation, microemulsion, hot melt extrusion technology, and supercritical fluid (SCF) technology. HPH is a reliable method that can be carried out in hot or cold conditions, suitable for both heat-sensitive and thermo-labile drugs [87].

Emulsification-ultrasonication is similar to HPH but involves ultrasonication after homogenization. Solvent diffusion and solvent emulsification evaporation methods use organic solvents and have limitations concerning solvent traces [89]. The film-ultrasonication method involves forming a thin layer of drug-lipid mixture and dispersing it in a heated aqueous phase during sonication [90]. Microemulsion method forms NLCs by precipitating microemulsion globules in cold water [91]. Hot melt extrusion technology and SCF technology are newer methods for NLC preparation, offering their own unique advantages [89].

The utilization of NLC for targeted drug delivery is being explored in the therapy of various types of cancer. For lung cancer, gene delivery using surface-modified NLCs has shown enhanced transfection and effectiveness in targeting cancer cells. Paclitaxel-doxorubicin co-loaded NLCs demonstrated efficacy against lung cancer cell lines and in vivo tumor models with reduced systemic toxicity [92].

In breast cancer, targeted drug delivery using NLCs has demonstrated potential in overcoming multidrug resistance and reducing systemic side effects. Various drug-loaded NLC formulations such as lapachone and doxorubicin co-loaded NLCs [93], thymoquinone loaded NLCs [94], C-substituted diindolylmethane derivatives as NLCs [95], have demonstrated enhanced anticancer efficacy in vitro and in vivo. Moreover, Doxorubicin and doxorubicin co-loaded with α-tocopherol succinate in NLCs exhibited enhanced efficacy in treating breast cancer in both in vivo tumors and cell lines [96]. Biochanin A NLCs demonstrated improved oral bioavailability by avoiding the reticuloendothelial system and demonstrated cytotoxicity against breast cancer cell lines, MCF-7 [97]. Co-administration of vitamin D-loaded NLCs enhances doxorubicin efficacy in MCF-7 cells indicating its potential effectiveness in breast cancer treatment when used alongside chemotherapeutic drugs [98].

For gastric and lymph cancer, NLCs loaded with drugs like etoposide, curcumin, doxorubicin, and vincristine have shown improved anti-tumor efficacy and reduced systemic toxicity compared to free drugs [99, 100]. In the case of brain cancer, NLC formulations of drugs such as vincristine and temozolomide [101], curcumin [102], and paclitaxel with transferrin [103] have demonstrated improved anti-tumor efficacy and targeted selection of brain tumor cell. Additionally, for the management of pain in cancer patients, tetrahydrocannabinol formulated as an NLC nasal spray has shown potential for improving bioavailability and onset of action [104].

### Hybrid LNPs

Hybrid LNPs have been developed as a unified approach consisting of a minimum of two types of materials to accomplish multiple functions or overcome the constraints of single-component nanomaterials, thus merging the strengths of the two distinct components [105]. Lately, a new type of drug delivery approaches known as hybrid lipid-polymeric nanoparticles has emerged, showing promising potential [106, 107]. Essentially, these nanoparticles feature a polymeric core that contains a therapeutic agent, surrounded by an inner lipid layer and an outer layer of PEGylated lipid [108]. The combination of lipid and polymer characteristics in these hybrid nanoparticles results in sustained release, exceptional stability, and high biocompatibility [108–110].

Researchers have utilized lipid-polymer hybrid nanoparticles to encapsulate a diverse variety of drugs, such as the clustered regularly interspaced short palindromic repeats (CRISPR)-associated protein 9 (CRISPR/Cas9) plasmids, sorafenib (SFN), DOX, raloxifene, and edelfosine, aiming for prolonged release, improved efficacy, and increased bioavailability [111–114]. To create these hybrid lipid-polymeric nanoparticles for drug delivery, it is crucial to ensure reproducibility and accurate control over nanoparticle characteristics. As a result, microfluidics has been widely employed in the fabrication of hybrid nanoparticles [115, 116].

Furthermore, various hybrid lipid-metal nanoparticles, including lipid-aluminum nanoparticles, lipid-coated silver nanoparticles (lipid-AgNPs), and liposome gold nanoparticles (LiposAu NPs), have been specifically engineered for a range of uses [117–121]. While metal nanoparticles are commonly utilized for biomedical labeling and probing, their effectiveness in studies based on *vivo* models is restricted due to poor colloidal stability and low cellular uptake. The application of lipid coatings to these metal nanoparticles is an ideal method for enhancing their biocompatibility, stability, and efficiency of cellular uptake [122]. Recent research has highlighted significantly improved stability of gold (Au) nanoparticles that are functionalized with phospholipids [123]. Additionally, LiposAu hybrid nanoparticles have demonstrated effective photothermal effects targeting cancer cells, offering a promising approach for cancer treatment [120].

### Cancer immunotherapy approaches

**Fig. 2:**
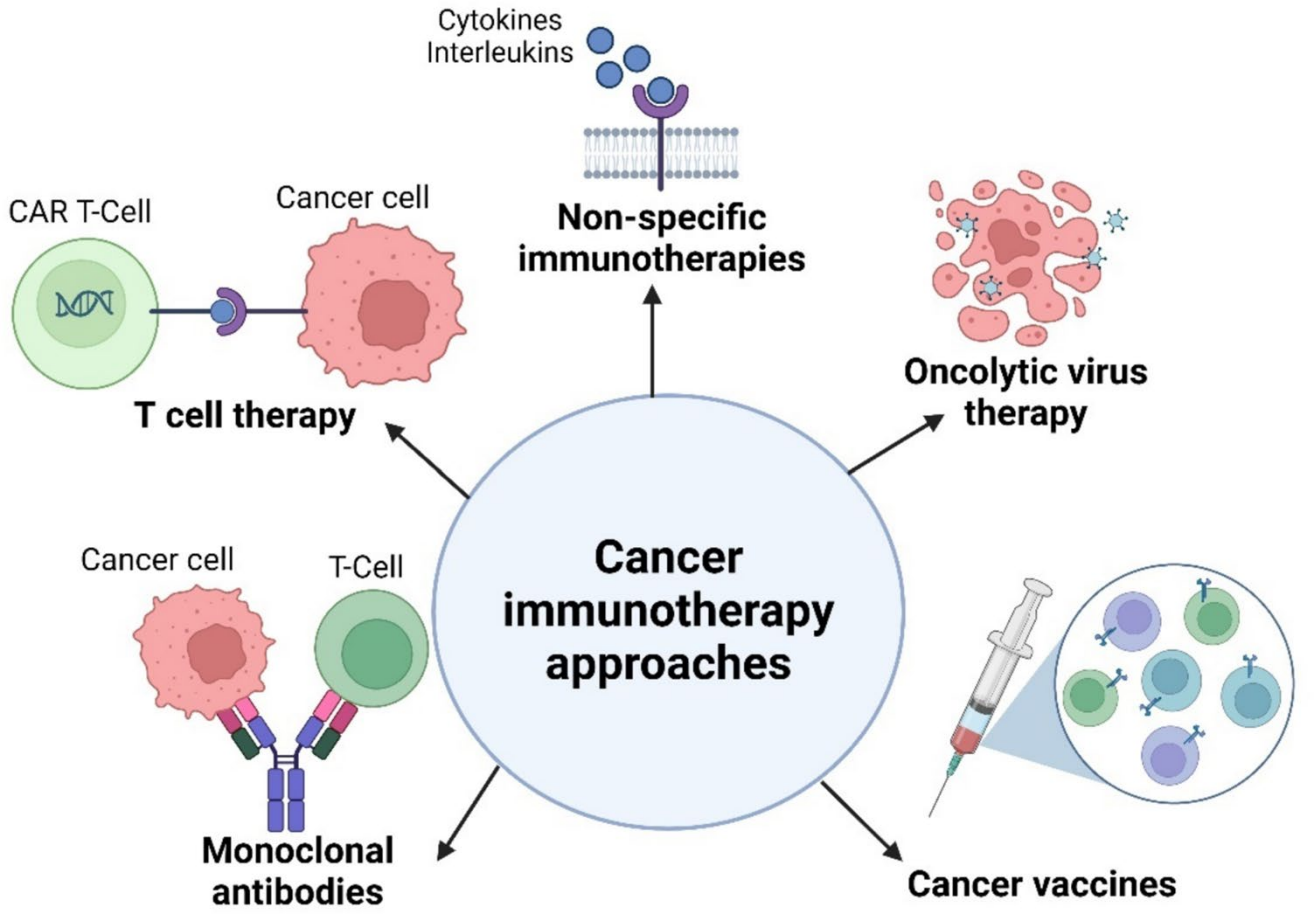
Cancer immunotherapy approaches

Recently, immunotherapy has evolved as a significant application of essential cancer immunology study. Unlike non-specific therapies such as radiotherapy or chemotherapy, cancer immunotherapy is frequently more targeted, training the immune system to recognize and remember tumor cells, offering an enduring approach post treatment. It works by guiding the immune system to target tumor cells through the recognition of tumor antigens and boosting the current anti-tumor immune responses. Current strategies encompass non-specific immunotherapy, monoclonal antibodies, immune checkpoints, cancer vaccines, oncolytic virus therapy, and T-cell therapy (Fig. 2) [124].

### Non-specific immunotherapies

Non-specific therapies support the immune system in attacking and eliminating cancer cells. These therapies, like chemotherapy and radiation, are typically administered alongside or after other cancer treatments. Among these strategies, cytokines which play an axial role in regulating both natural and adaptive immunity and have shown effective responses in preclinical mouse cancer models [125]. For instance, Interleukin-2 (IL-2) is approved for treating metastatic renal cancer and advanced melanoma, while interferon-alpha (IFN-α) is utilized to treat patients with conditions like AIDS-related Kaposi sarcoma, hairy cell leukemia, chronic myeloid leukemia, and follicular lymphoma. Clinical trials are also exploring the potential of other cytokines such as IL-21, IL-18, IL-15, IL-12, IL-7, and granulocyte–macrophage colony-stimulating factor [126, 127].

### Oncolytic virus therapy

Oncolytic virus therapy uses viruses with genetic modifications to selectively infect and replicate within malignant cells, resulting in immunogenic cancer cell death. The first FDA-approved virus for cancer therapy, talimogene laherparepvec (T-VEC or OncoVEX^GM−CSF^), was utilized to treat advanced melanoma in 2015 [128]. T-VEC, derived from a modified human herpes simplex virus 1 (HSV-1), promotes the recruiting and activation of antigen-presenting cells, initiating an anti-tumor immune response through viral oncolysis, with attenuated pathogenicity due to specific viral gene deletions (RL1 and US12) [129, 130]. Additionally, a research study demonstrated that intraperitoneal delivery of tumor-targeted oncolytic adenovirus (Ad-TD) with non-secreting IL-12 significantly improved the survival of hamsters diagnosed with pancreatic cancer [131]. Researchers are exploring various oncolytic viruses for different cancer types, as well as their combinations with other treatments such as immune checkpoint inhibitors or chemotherapy in clinical trials [132, 133].

### Cancer vaccines

While the development of effective therapeutic cancer vaccines is a major focus, their clinical application is challenging due to the low immunogenicity of the majority of tumor-associated antigens (TAAs). Cancer vaccines aim to recapitulate an immune response against tumor-specific antigens to combat cancer [134, 135]. In 2010, the FDA approved the therapeutic dendritic cell-based cancer vaccine, Sipuleucel-T, for treating metastatic castration-resistant prostate cancer [136]. DNA vaccines deliver plasmids containing TAAs coding sequences to induce the expression of these antigens, triggering an immune response [134].

Vaccines for viral infections linked to cancer, such as hepatitis B virus (HBV) and human papillomavirus (HPV), have shown promise in inhibiting associated cancers [137]. For instance, the HPV vaccine GX-188E enhanced immune response and promoted lesion regression in cervical intraepithelial neoplasia 3 (CIN3) patients [138]. Chronic infection with HBV has a significant impact on the development of hepatocellular carcinoma (HCC). Immunization against HBV has led to a notable decrease in the prevalence of HCC [139]. Additionally, personalized genomic vaccines are being explored, using the patient tumor genetic sequence to create customized vaccines [140]. Advanced bioinformatics and sequencing technologies may enable the accurate prediction of mutated genes’ immunogenicity, potentially leading to tailored cancer vaccines that can induce strong anti-tumor immunity [141].

### Monoclonal antibodies

Monoclonal antibodies (mAbs), also called therapeutic antibodies, were first created using the hybridoma technique in 1975 by Cesar Milstein and Georges Kohler. This groundbreaking innovation was honored with the Nobel Prize in Physiology or Medicine in 1984. It opened the door for the development of specific antibodies against a wide range of diseases [142, 143]. In 1997, the first monoclonal antibody, rituximab, received FDA approval for the treatment of B-cell non-Hodgkin’s lymphomas [136]. Antibody-based immunotherapy offers a targeted approach to treatment, utilizing the Fc region to engage with the host immune system and the Fv region to interact with specific targets. Over time, four main types of monoclonal antibodies (humanized, chimeric, murine, and human mAbs) have been developed [144].

Monoclonal antibodies (mAbs) can be characterized by their mechanisms of action. These mechanisms include antibody-dependent cell phagocytosis (ADCP), antibody-dependent cellular cytotoxicity (ADCC), and complement-dependent cytotoxicity (CDC) [145, 146]. Accordingly, they are utilized in three main ways: First, some mAbs label cancer cells to improve their recognition and destruction by the immune system, constituting a form of immunotherapy. Second, certain mAbs can hinder the function of abnormal proteins in cancer, serving as targeted therapy. Third, antibodies can unleash the immune system control on cancer growth and metastasis by targeting immune checkpoint pathways [147]. Immune checkpoint inhibitors, such as anti-CTLA-4 agents, anti-PD-1 agents, and anti-PD-L1 agents, have received FDA approval [148].

Several cancer drugs have been approved by the FDA in recent years. Ipilimumab (Yervoy), approved in 2011, targets CTLA-4 and is used for unremovable or metastatic melanoma, metastatic colorectal cancer, and renal cell carcinoma. It can be used in combination with nivolumab for various cancer types. Pembrolizumab (Keytruda), approved in 2014, targets PD-1 and is used for several sorts of cancer, including non-small cell lung cancer, melanoma, and urothelial carcinoma, and is used in combination with different chemotherapy regimens. Nivolumab (Opdivo), also approved in 2014 and targeting PD-1, is used for similar cancer types as pembrolizumab and can also be used in combination with other drugs. Other drugs like atezolizumab, avelumab, durvalumab, and cemiplimab have been approved for various indications, either as standalone treatments or in specific combinations for different types of cancer [124].

### T-cell therapy

Adoptive T -cell therapy is a missionary immunotherapy approach that exploits the tumor-fighting abilities of lymphocytes to target and revoke specific primary metastatic and cancer cells. This approach involves stimulating lymphocytes outside the body within a non-tolerant environment, subsequent to reintroducing activated T-cells into patients [149]. Various types of T-cells, including tumor-infiltrating lymphocytes, T-cells modified to express a cancer-specific T-cell receptor (TCR), and CAR-modified T-cells, are utilized in adoptive therapy [150].

Moreover, CAR-engineered T-cell therapy has advanced considerably, incorporating various generations of CARs with improved signaling domains to improve T-cell activity and target cell eradication Notably, CAR T-cell therapy has demonstrated promising results in clinical trials targeting B-cell antigens such as CD19, CD20, and CD22. FDA approval was granted to drugs like axicabtagene ciloleucel and tisagenlecleucel for treating lymphoblastic leukemia and non-Hodgkin lymphoma, respectively [124]. Nevertheless, the efficacy of CAR T-cell therapy with solid tumors is still a challenge because of the absence of specific antigens expressed on the many malignant cells [151].

## Nanoparticles for nucleic acid delivery in cancer immunotherapy

### DNA delivery

DNA vaccine-based cancer immunotherapy uses plasmid or chemically synthesized DNA to activate immune reactions against specific antigens, providing a robust strategy for involving the immune system in attacking cancer cells. [152]. In the initial mouse studies, the effectiveness of DNA plasmids in stimulating immune reactions against a variety of diseases was demonstrated, establishing DNA as a promising vaccination system [153]. Nevertheless, the initial clinical application of DNA vaccines encountered difficulties in eliciting significant immune responses, largely due to the limitations of naked DNA as a standalone vaccination approach and the challenges associated with delivering DNA into cells while minimizing off-target expression [153, 154]. To overcome these obstacles, researchers have investigated various physical and nanoparticle-based methods to enhance DNA delivery, such as employing liposomes, ionizable lipids, and polymer-LNPs [154].

Liposomes, particularly cationic liposomes, have shown promise in clinical development for cancer treatment but are limited by their potential toxicity, undesired immune reactions and the formation of clots, which impact the permissible administered dose [155–158]. Alternatively, the ionizable lipids development has provided a solution for promoting endosomal escape and enabling the processing of nucleic acids in the cytosol, offering a unique strategy for nucleic acid cancer immunotherapy [30, 159–161].

**Fig. 3:**
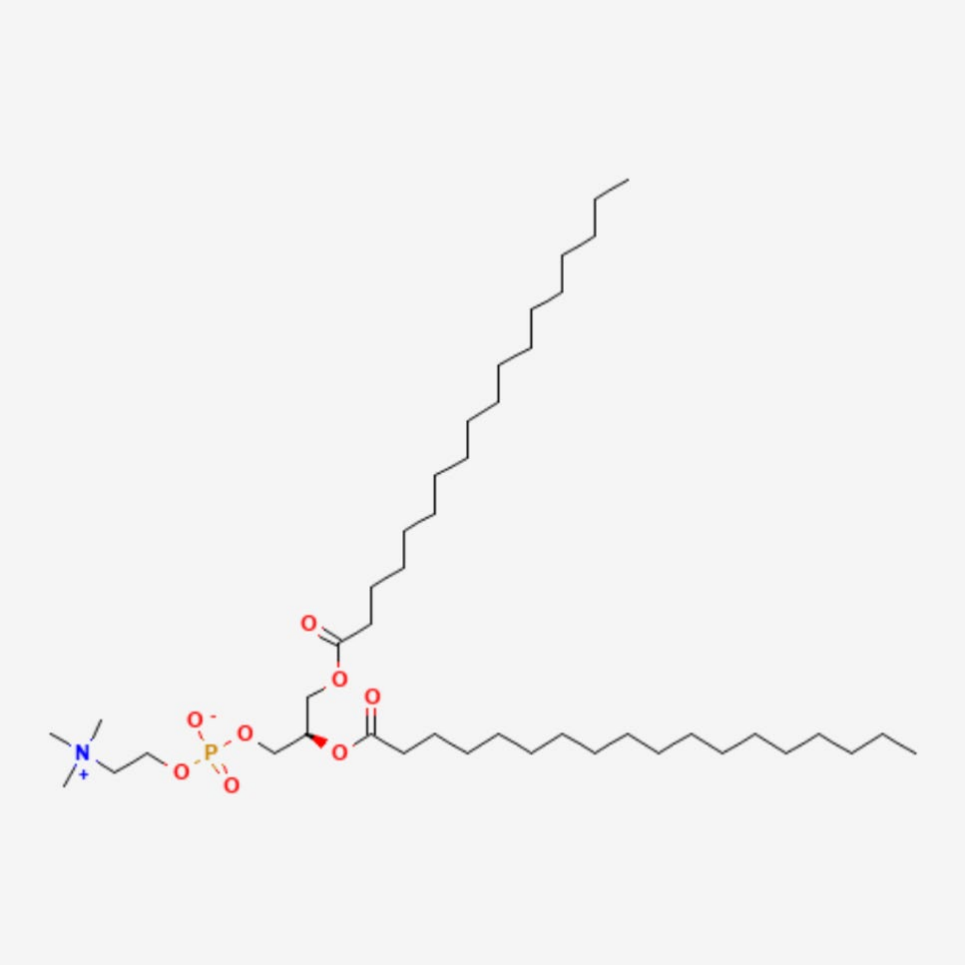
Chemical structure of 1,2-Distearoyl-sn-glycero-3-phosphocholine (DSPC)

**Fig. 4:**
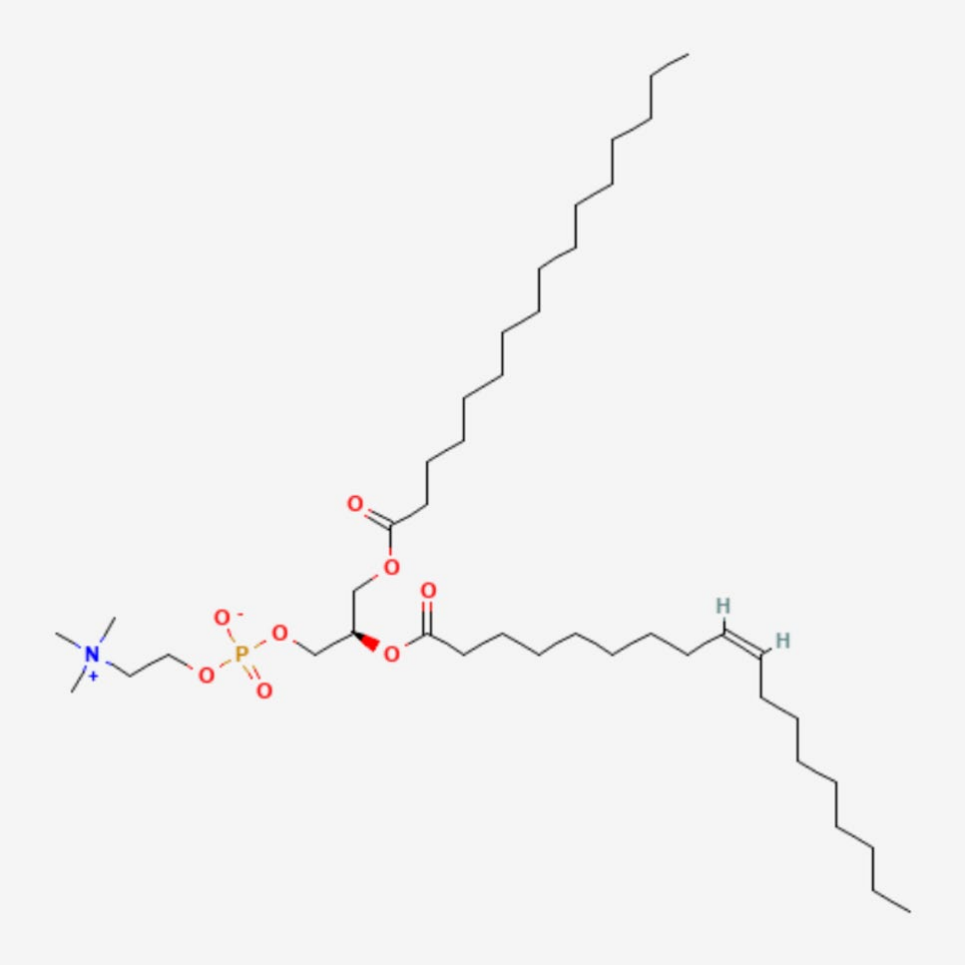
Chemical structure of 1-Palmitoyl-2-oleoyl-sn-glycero-3-phosphocholine (POPC)

**Fig. 5:**
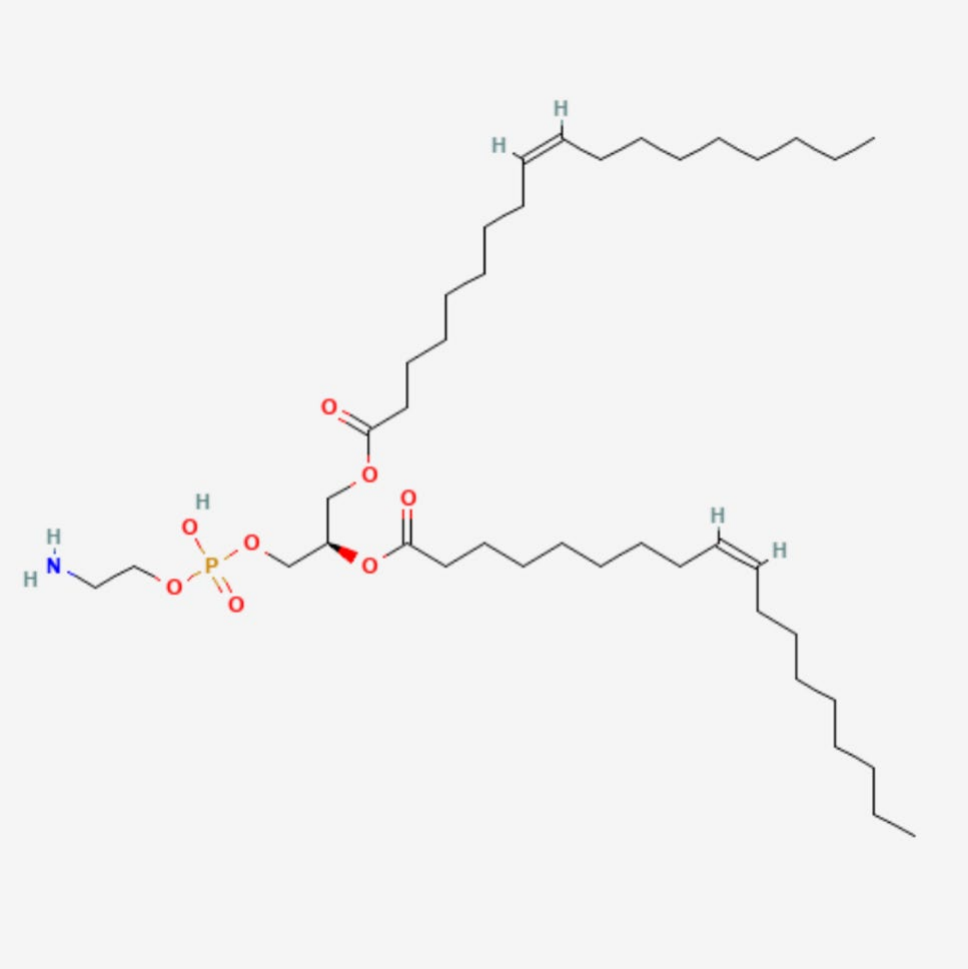
Chemical structure of 1,2-Dioleoyl-sn-glycero-3-phosphoethanolamine (DOPE)

**Fig. 6:**
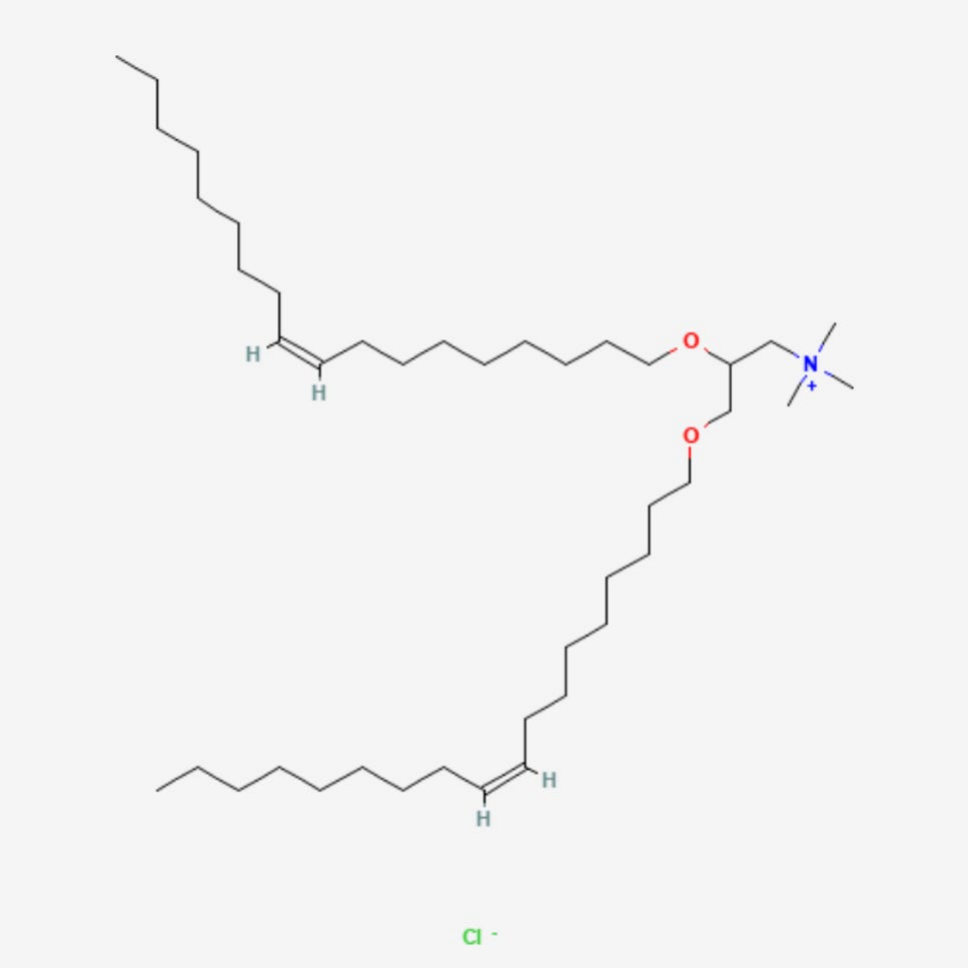
Chemical structure of Dioleoyl-3-trimethylammonium propane (DOTMA)

**Fig. 7:**
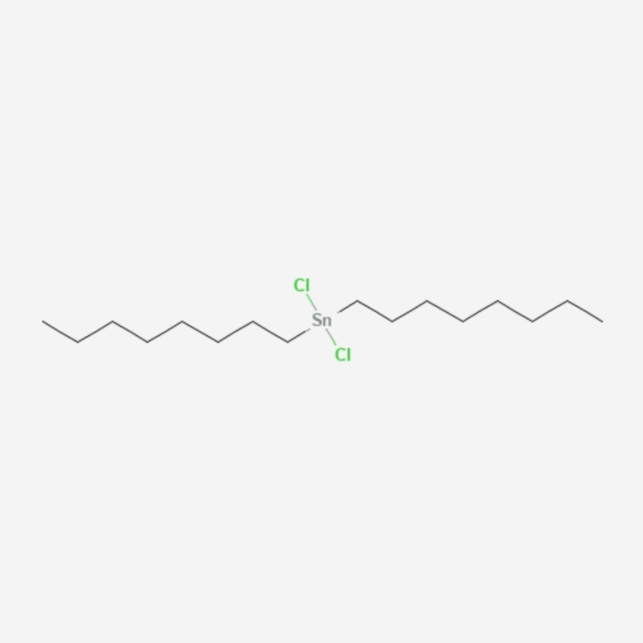
Chemical Structure of Dichlorodioctyltin (DOTC)

**Fig. 8:**
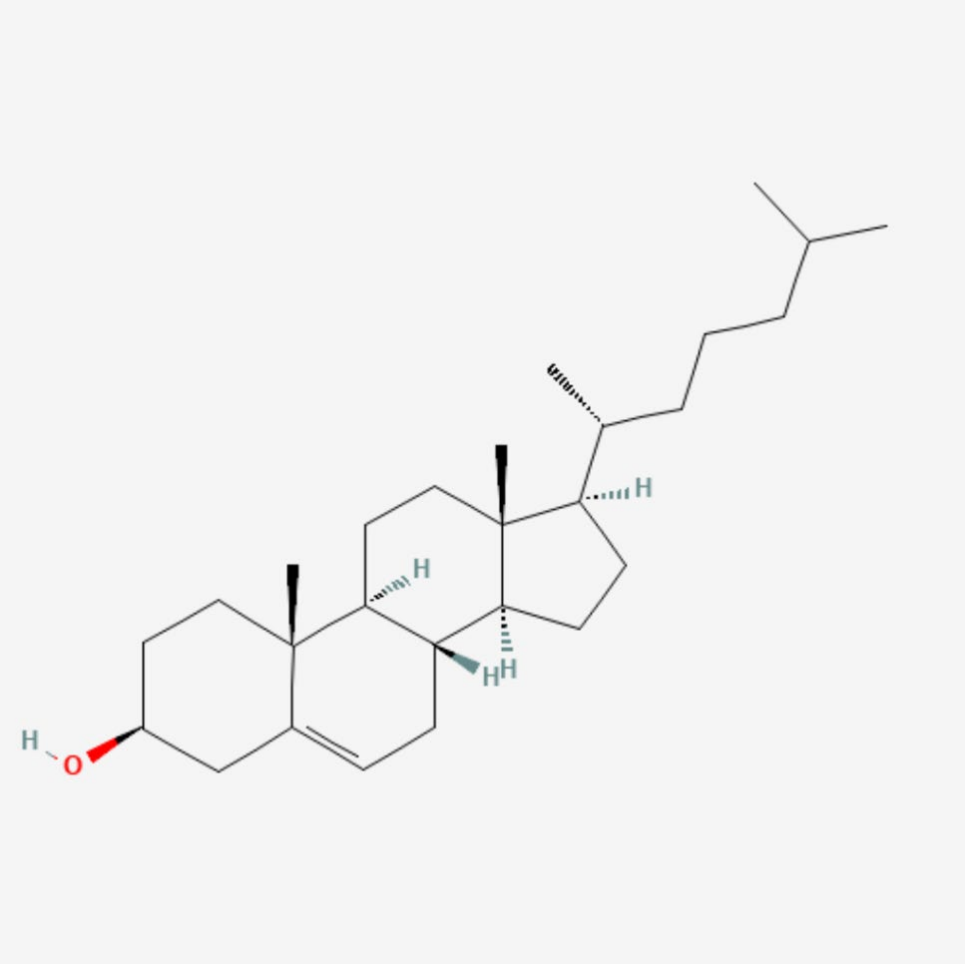
Chemical structure of cholesterol

LNPs typically consist of three components besides the ionizable lipid itself: a fusogenic helper phospholipid (such as DSPC (Fig. 3), POPC (Fig. 4), DOPE (Fig. 5), DOTMA (Fig. 6), DOTC (Fig. 7) [162, 163], cholesterol (Fig. 8) to enhance stability and membrane fusion [164, 165], and a lipid-anchored poly(ethylene glycol) (PEG) to prolong their circulation half-life and reduce non-specific protein binding [162]. These ionizable LNPs have been utilized in DNA cancer immunotherapy by encapsulating CpG oligodeoxynucleotides (ODNs), a TLR-9 agonist. Specifically, CpG-loaded nanoparticles were co-administered subcutaneously with tumor-associated antigens in mouse models of thymoma and melanoma [166].

The NPs demonstrated a tendency to accumulate in and be taken up by immune cells in lymph nodes, enhancing antigen-specific immune cell responses and cytokine/chemokine production, finally resulting in increased tumor eradication in a murine EG7-OVA tumor model [166]. While ionizable LNPs have proven effective in loading nucleic acids of comparatively small sizes such as short synthetic DNA, microRNA, siRNA, the encapsulation of larger cargoes such as plasmid DNA still a challenge [167–169]. Therefore, polymer-based nanoparticles including polyplexes [170–172], poly(beta-amino esters (PBAEs), and chitosan-based NPs [173, 174] have been created to efficiently compress plasmid DNA into NPs, enhancing transgene expression and demonstrating potential for cancer immunotherapy [175–177].

Cationic polymeric nanoparticles, including poly(l-lysine) (PLL) and polyethylenimine (PEI), have shown efficacy in condensing and delivering DNA, contributing to cancer immunotherapy efforts [178–180]. However, considerations for cytotoxicity and biocompatibility are essential when designing such delivery vehicles. A notable example involves the use of PEI for condensing IL-12-encoding pDNA, which demonstrated successful gene therapy for cancer in a murine model of osteosarcoma lung metastasis [181]. Additionally, PBAEs have been utilized for delivering leukemia-specific CAR pDNA to T-cells, showcasing their potential for promoting tumor regression in preclinical models [182, 183]. With these developments, DNA-based therapeutics continue to show promise in cancer immunotherapy [184], in addition to research focusing on the development of DNA nanoparticles for CAR T-cells or as adjuvants [183, 185, 186].

### mRNA delivery

Following the success of DNA-based therapeutics, mRNA has become a powerful tool in gene immunotherapy for cancer, presenting various distinct applications for further exploration in cancer treatment. mRNA therapeutics can present a promising substitute for DNA-based vaccines due to fewer delivery barriers, lower mutational risk, and transient expression, as well as the ability to induce protein translation in the cytosol, bypassing the need for nuclear entry [154, 187–189]. By encoding antigens, mRNA delivered to antigen-presenting cells stimulates cytotoxic T lymphocyte responses, making it a valuable tool for cancer immunotherapy [188].

NPs do a crucial function in overcoming the size, charge, and hydrophilicity limitations [187, 189–191] of naked mRNA, facilitating its uptake by target cells and subsequent intracellular delivery.

Ionizable LNPs have shown success in delivering mRN; in a specific instance, LNPs consisting of cholesterol, an ionizable lipid, a helper phospholipid, lipid-anchored PEG, and mRNA were engineered to stimulate the luciferase and erythropoietin expression upon systemic injection in BALB/c mice [49]. The research work used experimental design method to optimize a highly effective LNP initially designed for siRNA delivery and now deliver mRNA to the mice livers [49]. NP-based systems have proven effective for siRNA delivery. To fully optimize their unique properties such as biocompatibility and low cytotoxicity, ongoing efforts are focused on optimizing formulation parameters, improving targeting efficiency, and enhancing cellular uptake. LNPs, polymers, and hybrid nanoparticles are among the most commonly used carriers for siRNA loading [192].

In another study, multilamellar ionizable LNPs were utilized to transport glycoprotein 100 (gp100) and tyrosine-related protein 2 (TRP2) tumor self-antigen mRNAs to antigen-presenting cells in order to prompt a cytotoxic CD8 T cell response. The subcutaneous administration of the LNPs resulted in reduced volumes in tumor, produced potent CD8 + activation, and prolonged survival in the tumor model system B16F10 [160]. These LNPs demonstrated the ability to transfect macrophages, neutrophils, and dendritic cells, indicating their potential for delivering mRNA to diverse immune cell types [193].

Moreover, Polymer-based systems offer a diverse range of structures for enhancing the encapsulation, stability, and delivery of mRNA [194]. PBAEs, known for their pH responsiveness and biocompatibility, have been engineered (including PEG-lipids and novel PBAE structures) to enhance serum biodistribution and stability, leading to selective mRNA expression in murine lungs and successful mucosal immunization in preclinical studies [195–198].

NPs also prove to be valuable for direct mRNA delivery to T-cells without requiring electroporation, presenting an attractive alternative to ex vivo cell engineering methods for cancer vaccination. For example, an initial strategy for an mRNA cancer vaccine involved the use of PEGylated histidine-rich polylysines combined with l-histidine-(N,N-di-n-hexadecylamine)ethylamide (HDHE) and cholesterol liposomes, which were designed to deliver mRNA encoding the human melanoma antigen MART1 to T-cells. This delivery system, known as histidylated lipopolyplexes, induced strong immune responses in a melanoma model, leading to reduced tumor volume and lung metastases relative to control mice [199]. Additionally, PBAE -NPs coated with antibodies CD3 or CD8 were found to significantly improve ex vivo T cell transfection relatively to non-targeted PBAE NPs [200]. This system has also been utilized to deliver distinct types mRNAs, including megaTAL nuclease mRNA to knockout endogenous T cell receptors and mRNA encoding the Foxo13A transcription factor to guide T-cells toward a central memory phenotype [200]. Clinical translation of mRNA vaccines, especially those delivered using NP systems, is now underway with promising preliminary results. Negatively charged or neutral LNP-mRNA complexes have shown good tolerability and dose-dependent immune responses in early-phase clinical trials, emphasizing the potential of NPs in advancing mRNA-based cancer immunotherapy from preclinical success to clinical applications [201].

### Gene editing

CRISPR/Cas9 has become a potent instrument for comprehending and addressing the genetic origins of diverse diseases [202, 203]. In cancer immunotherapy, CRISPR has been utilized for producing disease models [204–206], identifying targets [207], and engineering immune cells [208–211]. CRISPR/Cas9 can be administered nucleic acids or as ribonucleoprotein [212, 213].

The CRISPR/Cas9 system has the potential to face limitations, such as off-target editing and the activation of the previously existing immune responses to the Cas9 protein. Delivering Cas9 protein and mRNA provides temporary expression of protein, which could be advantageous due to the potential risks of continuous expression [214–216]. Yet, successful efforts to package Cas9 ribonucleoprotein into NPs have primarily focused on localized delivery, while systemic administration has been effective for Cas9 mRNA [216–221].

Ju et al*.*, proposed a novel metabolic engineering approach targeting lactate dehydrogenase A (LDHA) in the tumor microenvironment. They developed a cationic lipid nanoparticle formulation for editing the LDHA gene. They evaluated the delivery efficacy of their LNPs formulations by using plasmid DNA that encoded green fluorescence protein. They found that the delivery efficiency depended on the proportions of three components (cholesterol or its derivative, cationic lipid, and a fusogenic lipid). One specific LNPs formulation, F3, was combined with plasmid DNA to create the lipoplex pCas9-sgLDHA/F3, enabling gene editing in cell cultures. It was effective in editing the LDHA gene. Additionally, it activated immune responses in vitro and showed a synergistic anti-tumor impact when combined with an anti-PD-L1 antibody in vivo [222]. In another study, the drawbacks of low editing efficacy and potential toxicity in the existing delivery systems for CRISPR-Cas9 technology in cancer therapies are covered. An amino-ionizable lipid is utilized to create a safe and effective LNPs for delivering Cas9 mRNA and sgRNAs. In aggressive orthotopic glioblastoma, these CRISPR-LNPs achieved up to 70% gene editing in vivo, leading to 50% inhibition of tumor growth, tumor cell apoptosis, and a 30% enchantment in survival. Additionally, cLNPs were modified for antibody-targeted delivery to reach disseminated tumors. Intraperitoneal injections of EGFR-targeted sgPLK1-cLNPs resulted in targeted intake by spread ovarian tumors, enabling up to about 80% gene editing in vivo, inhibiting tumor growth, and increasing survival by 80%. This breakthrough in the capability to disrupt gene expression in tumors in vivo opens promising innovative pathways for cancer therapy and investigation [223].

## LNP for nucleic acid delivery in cancer treatment

### miRNA delivery

MicroRNAs (miRNAs) are small, non-coding RNA molecules approximately 22 nucleotides long. They operate post-transcriptionally to influence gene expression. More than 2500 miRNA sequences have been discovered in humans, regulating the expression of genes in mammals. Additionally, over 60% of human protein-coding genes have been observed to harbor at least one binding site for miRNAs, highlighting the important role of miRNA-mediated regulation in the human genome [224, 225]

Certain miRNAs are specifically expressed or reduced in particular types of cancer. Research has shown that miRNAs can function as oncogenes (known as oncomiRs) or tumor suppressors. Furthermore, they are utilized as therapeutic tools to silence overactive genes and reverse the characteristics of cancer cells. Many studies have concentrated on combatting cancer by either blocking oncomiRs or increasing the expression of tumor suppressor miRNAs, presenting a hopeful approach for cancer treatment [226].

LNPs have proven to be effective carriers for delivering miRNA molecules. For example, in the context of breast cancer (BC), Liu et al*.* engineered a LNPs-based system for the simultaneous delivery of paclitaxel (PTX) and miR-200c [47]. Another investigation by *Hayward *et al*.* utilized LNPs to transport miRNA125a-5p to 21MT-1 cells in order to silence HER2. They enhanced the targeting of cancer cells, endosomal escape, and uniform distribution of miRNA in the cytoplasm by coating LNPs with hyaluronic acid as a targeting agent. Furthermore, the HER2 proto-oncogene was suppressed at both the transcriptional and translational levels, as confirmed by qRT-PCR and western blot analyses [227].

Recently, Dinami et al*.* employed a miRNA-based approach to inhibit the telomeric protein Telomere repeat binding factor 2 (TRF2), inducing cell death in triple-negative breast cancer (TNBC). They developed LNPs with a lipid mixture of DSPC/CHOL/DLODAP/PEG2000‐Cer16 (25/45/20/10 w/w) to deliver the miRNA. This approach effectively decreased TRF2 levels and inhibited growth in various cancer cell lines. Silencing TRF2 with miR‐182‐3p led to DNA damage at pericentromeric and telomeric sites, causing genomic instability. Furthermore, the study revealed that LNPs encapsulating miR‐182‐3p reduced tumor volume in vivo in different TNBC models, including those resistant to Olaparib. Additionally, the LNPs‐miR‐182‐3p formulation demonstrated the ability to penetrate the blood–brain barrier, highlighting its potential in combating brain metastases [228].

### circRNA delivery

CircRNAs are a recently identified subclass of non-coding RNA molecules formed during RNA transcript maturation. Initially considered splicing byproducts, they are now acknowledged as functional RNA molecules with distinct expression patterns in various tissues and cell types. CircRNAs are derived from a wide array of genes and play roles in processes associated with cancer development and progression [2]. Moreover, there is increasing evidence suggesting the potential role of circRNAs in regulating natural defenses against tumors [229, 230].

While circRNAs are being explored as potential tools for immunotherapy, their instability presents a significant obstacle. One approach to addressing this challenge involves encapsulating circRNAs in LNPs, providing protection against degradation, enhancing uptake by cells, and facilitating entry into the cellular machinery through endosomal pathways. This enables circRNAs to exert their anti-tumor effects in living organisms. Furthermore, circRNA-loaded nanoparticles can be designed to activate both innate and adaptive immune responses. For instance, a new vaccine platform utilizing LNPs that encapsulate circRNAs encoding antigens has been developed to take advantage of the prolonged protein translation capability of circRNAs and to establish a context that stimulates the innate immune system. This approach has demonstrated enhanced generation of potent, cytotoxic, antigen-specific T-cell responses and superior anti-tumor effects in various mouse tumor models, including the “immune-desert” B16 orthotopic melanoma [231].

Another study has shown that intratumoral delivery of circular mRNA, encoding a mixture of cytokines via LNPs, promotes both local and systemic anti-tumor immune responses and enhances the effectiveness of anti-programmed cell death protein 1 (PD-1) antibody therapy in a model of mouse tumors with a similar genetic makeup [232]. Additionally, circRNA has the potential to act as a powerful enhancer of adaptive anti-tumor T-cell responses within the context of vaccinations [233]. However, despite these promising developments, there are significant challenges that need to be overcome before nanoparticle-based circRNA immunotherapy can be translated into clinical treatment for a range of cancers.

### siRNA delivery

SiRNAs, which are about 25 nucleotides long, are a type of double-stranded RNA involved in RNA interference. In the cytoplasm, dedicated enzymes process siRNAs, which then bind to complementary mRNA sequences, leading to mRNA degradation and blocking protein production. The FDA has approved four siRNA-based treatments, highlighting the potential effectiveness and safety of this approach [234]. SiRNA-based RNA interference is seen as a promising method for selectively silencing genes, especially tumor-specific ones [235, 236]. siRNAs specifically target a single mRNA sequence, reducing off-target effects that can affect multiple genes. However, challenges related to preventing degradation and effectively delivering siRNA persist, which can be partly addressed by using LNPs as carriers [237].

In recent years, siRNAs have emerged as powerful cancer therapeutics due to their ability to silence genes involved in tumor formation and spread [238]. Yet, delivering siRNAs to their targets has been a significant challenge [239]. LNPs have become a promising method for effectively delivering siRNAs, as they protect the siRNAs from degradation and enable selective delivery to target cells, increasing their availability and reducing off-target effects. Consequently, numerous studies have investigated LNPs as delivery systems for siRNA-based therapies [240–244]. Kiaie et al*.* developed siP2X7-LNPs, a lipid nano carrier for delivering siRNA targeting the P2X7 receptor, showing promising therapeutic efficacy in a triple-negative breast cancer cell line [245].

### mRNA delivery

mRNA, a single-stranded nucleic acid sequence derived from DNA transcription, carries genetic information from the nucleus to the cytoplasm where it is translated into proteins. This molecule has therapeutic potential due to its ease of design, minimal off-target effects, and transient expression, making it unlikely to cause genetic mutations. The success of mRNA-based vaccines during the recent pandemic has highlighted its robustness and safety, leading to exploration of new therapeutic applications [246].

Researchers have enhanced mRNA stability, immunogenicity, and translation efficacy, paving the way for potential use in cancer therapy. By delivering mRNAs encoding for various targets such as tumor suppressor proteins, antigens, or cytokines, mRNA therapeutics have the potential to inhibit cancer cell proliferation and enhance immune responses. Additionally, mRNA-encoded genome-editing proteins show promise in improving treatment efficacy. LNPs have been identified as an efficient platform for mRNA delivery, further supporting its potential as a versatile therapeutic tool [246].

Progress in drug delivery systems has advanced the preclinical development of mRNA therapeutics, positioning them as a novel drug class [187]. LNPs have shown clinical promise for delivering mRNA [247, 248], for example, El-Mayta and colleagues recently demonstrated an mRNA encapsulation efficiency of 92.3% for C12-200 mRNA-LNPs with specific ratios of ionizable lipid, helper lipid, cholesterol, and lipid-PEG [249].

Zhang et al. conducted a study on the development of PTX amino lipid (PAL)-derived NPs, aiming to combine chemotherapy and p53 mRNA to create innovative treatment strategies for TNBC. The PAL p53 mRNA-LNPs exhibited high PTX loading capacity (94.7% ± 6.8%) and mRNA encapsulation efficiency (88.7% ± 0.7%), outperforming clinically used drugs like Abraxane® and Lipusu®. These LNPs effectively demonstrated the combined cytotoxic effect of PTX and p53 mRNA on TNBC cells and showcased anti-tumor efficacy in TNBC mouse models. This study introduces a promising platform for integrating chemotherapy with personalized medicine to treat TNBC [250].

## The potential of LNP in cancer immunotherapy

LNP-based treatments have been widely employed in both preclinical and early clinical trials within the field of cancer immunotherapy. Currently, LNPs are commonly engineered to transport multiple nucleic acids as cancer immunotherapeutic agents that target antigen-presenting cells (APCs) to stimulate and activate antigen-specific T-cell-mediated immune responses. Among various types of LBPs, positively charged cationic LNPs offer significant advantages in the encapsulation and delivery of drugs, considering the inherent molecular charge of most involved nucleic acids. For instance, Chaudhuri et al*.* have introduced lysinylated cationic amphiphiles covalently grafted with mannose-mimicking shikimoyl and quinoyl groups in the head group region to achieve stable and efficient DNA loading and long-term immune responses [251]. In another research group, they developed an RNA–liposomal (RNA-NPs) cancer vaccine with personalized tumor-derived mRNA encapsulated in DOTAP nanoparticles [252]. Despite the advantages of effective encapsulation, the surface charge of cationic LNPs has hindered their further use. This is because positively charged NPs have been found to impact lymphatic transport and tissue permeation after injection, leading to their immobilization within the negatively charged extracellular matrix, thus causing hemolysis and platelet aggregation [253].

Consequently, several studies have started to focus on the in vivo localization of cationic LNPs. For instance, Kranz et al. managed to significantly reduce the overall charge of mRNA and LNPs (consisting of DOPE and DOTMA) by adjusting the lipid:mRNA ratio [201]. Their findings indicated that near-neutral mRNA–liposome systems with a slightly positive charge could be localized within the spleen instead of aggregating in areas such as the heart and lungs. The interaction between loaded mRNA and various LNP delivery platforms was systematically assessed to determine the most effective combination for the uptake and expression of the encoded antigen by DC populations. As a result, the mRNA utilized in this study has the potential to activate Toll-like receptor 7 (TLR7) on DCs, leading to the secretion of IFN and desired anticancer responses. An initial phase I trial based on this study demonstrated that IFNα and strong antigen-specific T-cell responses could be induced at low doses in patients with advanced malignant melanoma [37].

LNPs have been utilized in the delivery of various FDA-approved drugs to address cancer-related conditions. Doxil, a liposomal doxorubicin approved in 1995, acts as a topoisomerase II inhibitor and is used in the treatment of leukemias, multiple myeloma, Hodgkin’s lymphoma, and various other cancers. Another approved drug, DaunoXome (liposomal daunorubicin), also functions as a topoisomerase II inhibitor and has been employed in the treatment of various cancers, including HIV-associated Kaposi’s sarcoma. Additionally, Marqibo, a liposomal vincristine approved in 2012, acts as a tubulin inhibitor and is used in the treatment of lymphoma, leukemia, melanoma, and brain cancer. Vyxeos, a liposomal formulation containing daunorubicin and cytarabine, was approved in 2017 for the treatment of acute myeloid leukemia (AML) as a topoisomerase II inhibitor and antimetabolic agent. These examples showcase how lipid-based nanoparticles have contributed to the delivery of therapeutic agents for the management of cancer and related conditions [37].

Liposomes with pH-sensitive dextran functionality have been extensively used to expose antigens to antigen-presenting cells (APCs) and to act as vaccine adjuvants to boost specific immune responses to antigens [254, 255]. These liposomes have been observed to be efficiently taken up by dendritic cells, enabling the cytosolic delivery of ovalbumin, which accelerates antigen-specific immune reactions and impedes the progression of cancer [256]. Additionally, the incorporation of CpG-ODNs (TLR-9 agonist) and 3,5-didodecyloxybenzamidine (adjuvant) into liposomes has been shown to amplify dendritic cell-mediated cytokine production, thus augmenting antigen-specific immunity [257]. Another type of LNPs, micelles, also serve as effective delivery systems for antigens and adjuvants to enhance the efficacy of vaccines. Polymeric hybrid micelles have been employed to deliver CpG-ODN and Trp2, creating a nanovaccine that targets lymph nodes, enhances cargo accumulation in dendritic cells, triggers CD8 + T-cell-mediated immune responses, and boosts cancer suppression in melanoma [258].

## Clinical status and marketed products

The clinical translation of LNPs as delivery vehicles for nucleic acid therapies has transformed modern medicine. Several lipid-based formulations have gained regulatory approval and are widely used in clinical practice. One of the earliest successes was **Doxil®**, the FDA-approved liposomal formulation of doxorubicin. Doxil is indicated for the treatment of ovarian cancer, Kaposi’s sarcoma, and multiple myeloma. By encapsulating doxorubicin in PEGylated liposomes, Doxil reduces cardiotoxicity and improves drug circulation time compared to the free drug. Another major milestone was the approval of **Onpattro® (Patisiran)**, the first siRNA drug delivered using LNPs. Onpattro treats hereditary transthyretin-mediated amyloidosis by silencing the mutant TTR gene, demonstrating the potential of RNA interference therapeutics.

More recently, the emergence of mRNA vaccines for COVID-19 has showcased the critical role of LNPs in vaccine development. Both **Comirnaty® (Pfizer–BioNTech)** and **Spikevax® (Moderna)** employ LNPs to protect and deliver mRNA encoding the SARS-CoV-2 spike protein. These vaccines have been instrumental in controlling the pandemic, highlighting LNPs’ ability to enhance nucleic acid stability, facilitate cellular uptake, and promote endosomal escape for effective protein expression.

Looking forward, lipid-based nucleic acid delivery systems hold great promise across many areas of medicine. Personalized medicine approaches are leveraging LNPs to deliver patient-specific mRNAs or gene-editing tools such as CRISPR-Cas9, enabling customized therapies for genetic and acquired diseases. In oncology, new LNP formulations are being developed that combine nucleic acids with immunostimulatory agents to enhance targeted cancer immunotherapy. Efforts are also underway to create next-generation vaccines that are thermostable and orally bioavailable, which would significantly improve global vaccine distribution and administration.

In addition, the delivery of gene-editing components like Cas9 mRNA and guide RNAs via LNPs is an exciting area that may provide curative treatments for monogenic disorders by enabling precise genome modifications. Furthermore, multifunctional LNPs capable of co-delivering nucleic acids along with small molecule drugs or immune modulators are being explored to achieve synergistic therapeutic effects. Despite ongoing challenges related to targeting specificity, immune clearance, and scalable manufacturing, lipid-based delivery platforms are poised to become foundational tools for a wide range of nucleic acid therapeutics in clinical settings. Table 2**.** summarizes clinically approved nanomedicine platforms for therapeutic delivery of small molecules, siRNA, mRNA, and viral vectors. It includes key formulation types such as PEGylated liposomes, LNPs, and bioviral vectors, along with their APIs, approved indications, and year of regulatory approval. These examples highlight the translational success of nanoparticle technologies in cancer therapy, infectious disease, and genetic disorders.

**Table 2:** FDA-approved nanoparticle and related advanced delivery formulations

Drug Name	API / Modality	Delivery System Type	Indication	Year Approved	Reference
Doxil®	Doxorubicin	PEGylated Liposome	Ovarian cancer, multiple myeloma, KS	1995	Barenholz, [12]
Onpattro®	Patisiran (siRNA)	Ionizable LNPs	Hereditary transthyretin amyloidosis	2018	Akinc et al. [25]
Comirnaty®	BNT162b2 (mRNA—Pfizer)	PEGylated Ionizable LNPs	COVID-19 vaccine	2020	Fortner and Schumacher [259]
Spikevax®	mRNA-1273 (Moderna)	PEGylated Ionizable LNPs	COVID-19 vaccine	2020	Baedeker et al. [260]
Leqvio®	Inclisiran (siRNA)	Solid LNPs	Hypercholesterolemia	2021	Lamb [261]
Imlygic®	OncoVEX^GM − CSF (HSV-1 virus)	Oncolytic virus (biovector)	Advanced melanoma	2015	Greig [262]

### Future prospects

The future prospects of LNPs in nucleic acid delivery are exceptionally promising and rapidly evolving. Advancements in lipid chemistry and nanoparticle engineering continue to improve targeting specificity, delivery efficiency, and biocompatibility. Researchers are developing LNPs that can cross challenging biological barriers such as the blood–brain barrier, enabling treatments for neurological disorders. Additionally, next-generation LNPs are being designed for controlled and stimuli-responsive release, allowing precise spatiotemporal delivery of therapeutic nucleic acids. The integration of artificial intelligence and machine learning is accelerating the discovery of novel lipid formulations optimized for specific nucleic acid cargos and disease targets.

Moreover, combining LNPs with other therapeutic modalities, such as immune checkpoint inhibitors or gene-editing technologies, holds potential to create highly effective combination therapies for cancer and genetic diseases. Personalized medicine will benefit from modular LNP platforms that can be quickly adapted to deliver patient-specific mRNA or gene-editing tools, facilitating rapid responses to emerging diseases or mutations.

Despite challenges related to large-scale manufacturing, stability, and immunogenicity, ongoing research and clinical trials are steadily overcoming these barriers. With continued interdisciplinary collaboration and technological innovation, lipid-based nucleic acid delivery systems are expected to expand their clinical applications beyond vaccines to encompass treatments for rare genetic disorders, infectious diseases, autoimmune conditions, and beyond, marking a new era in precision medicine and therapeutic intervention.

## Conclusion

In conclusion, this review highlights the significant role of LNPs in advancing cancer immunotherapy through the efficient delivery of nucleic acid therapeutics. The versatile nature of LNPs enables the effective encapsulation and targeted delivery of various nucleic acid molecules, fostering innovative and precise approaches for cancer treatment. From targeting tumor antigens to modulating immune responses, LNPs have demonstrated promising results in both preclinical and early clinical studies [263]. Looking forward, emerging trends such as personalized LNPs tailored to individual patient profiles hold great promise for enhancing therapeutic efficacy and minimizing adverse effects. Combination therapies that integrate LNP-based mRNA delivery with immune checkpoint inhibitors are showing synergistic potential to amplify anti-tumor immune responses. Additionally, the integration of artificial intelligence (AI) and machine learning in the design and optimization of LNP formulations is accelerating the development of more effective and safer delivery systems by predicting optimal lipid compositions and physicochemical properties. These advances underscore the dynamic evolution of LNP technology as a cornerstone of next-generation cancer immunotherapies. By harnessing personalized medicine, combination strategies, and AI-driven design, LNPs are poised to revolutionize the landscape of cancer treatment, offering new hope for patients through improved precision and efficacy of nucleic acid-based therapies.

## Data Availability

No datasets were generated or analysed during the current study.

## Abbreviations

siRNA: Small Interfering RNA
mRNA: Messenger RNA
FDA: Food and Drug Administration
CRISPR: Clustered Regularly Interspaced Short Palindromic Repeats
TLR7: Toll-Like Receptor 7
APCs: Antigen-Presenting Cells
DC: Dendritic Cells
TNBC: Triple-Negative Breast Cancer
IFN: Interferon
PTX: Paclitaxel
PEG: Polyethylene Glycol
DOPE: Dioleoylphosphatidylethanolamine
DOTAP: 1,2-Dioleoyl-3-Trimethylammonium Propane
LNPs: Lipid Nanoparticles
TLR-9: Toll-Like Receptor 9
CpG-ODNs: Cytosine-Phosphate-Guanine Oligodeoxynucleotides
RNA-NPs: RNA Nanoparticles
mRNA-LNP: MRNA Encapsulated in Lipid Nanoparticles
p53: Tumor Suppressor Protein p53
PEG-Lipid: Polyethylene Glycol Lipid
miRNA: MicroRNA
circRNA: Circular RNA
PTX: Paclitaxel
HIV: Human Immunodeficiency Virus
AML: Acute Myeloid Leukemia
CML: Chronic Myeloid Leukemia
BCL-2: B-Cell Lymphoma 2
HGF: Hepatocyte Growth Factor
VEGF: Vascular Endothelial Growth Factor
IC50: Half-Maximal Inhibitory Concentration
Doxil: Liposomal Doxorubicin
DaunoXome: Liposomal Daunorubicin
VSV: Vesicular Stomatitis Viru
